# Refining Pharmacologic Research to Prevent and Treat Spontaneous Preterm Birth

**DOI:** 10.3389/fphar.2017.00118

**Published:** 2017-03-15

**Authors:** Tracy A. Manuck

**Affiliations:** Department of Obstetrics and Gynecology, University of North Carolina-Chapel HillChapel Hill, NC, USA

**Keywords:** spontaneous preterm birth, pharmacogenomics, neonatal morbidity, 17-alpha hydroxyprogesterone caproate, tocolysis, indomethacin, personalized medicine

## Introduction

Preterm birth (PTB), delivery prior to 37 weeks' gestation, is the leading cause of mortality among non-anomalous neonates. Survivors carry an increased risk for lifelong intellectual, physical, and social disabilities compared with their term counterparts (Russell et al., 2007; Bodeau-Livinec et al., 2008; Vohr, 2013; Manuck et al., 2014a, 2016b; Natarajan and Shankaran, 2016). In the US alone, more than 450,000 babies are born too soon and ~25,000 die as a result (Hamilton et al., 2015). Approximately two-thirds of all PTBs are spontaneous PTB (SPTB), and occur following preterm premature rupture of membranes, cervical insufficiency, and/or uterine contractions leading to cervical dilation. To reduce the burden of SPTB, interventions must target both prematurity prevention prior to the onset of symptoms and acute treatment once the process of acute preterm labor has begun.

Research efforts to develop treatments to improve neonatal outcomes have met some success [e.g., 17-alpha hydroxprogesterone caproate for recurrent SPTB prevention (Meis et al., 2003), antenatal corticosteroid treatment to prevent sequelae of prematurity (Roberts and Dalziel, 2006; Gyamfi-Bannerman and Thom, 2016)]. However, our ability to effectively prevent and treat SPTB remains limited. Of the available treatment options, significant inter-individual variation is appreciated. The reasons for this response variation are poorly understood and represent a critical knowledge gap contributing to thousands of SPTB every year. Refinement of patient selection for available drugs, or changing the form or dose of medication has the potential for large impact on therapeutic efficacy. Here we highlight two examples of medications currently used to reduce SPTB and discuss how cutting edge approaches may improve outcomes (Table 1).

**Table 1:** **Listed is a summary of example medications for prevention and treatment of spontaneous preterm birth, along with current and future approaches to optimize response to these therapeutic interventions**.

**Indication**	**Example medication**	**Optimizing response to therapy**	
		**Current**	**Future**
Prevention of prematurity	17-OHPC	- Predict response based on clinical risk factors	- Determine optimal dose based and likelihood of response based on maternal and/or fetal genotype, methylome, transcriptome -Interrogate gene-environment interactions as etiology in variable response -Evaluation of effects of concominant drug administration on 17-OHPC levels
Treatment of acute preterm labor	Indomethacin	- None	- All of the above, plus: -Application of nanotechnology to minimize fetal exposure while maximizing tissue-specific effects

*17-OHPC, 17-alpha hydroxyprogesterone caproate*.

## Prevention of recurrent SPTB with intramuscular progesterone

A personal history of SPTB is the strongest clinical risk factor, conferring a 2- to 4-fold risk for SPTB (Hamilton et al., 2015). In a large multi-center randomized controlled trial conducted by the NICHD Maternal-Fetal Medicine Units Network, Meis et al. studied weekly intramuscular 17-alpha hydroxyprogesterone caproate (17-OHPC) vs. placebo in women with a history of a prior SPTB. The rate of recurrent SPTB <37 weeks gestation was reduced from 55% in the placebo group to 36% in the 17P group (RR 0.66, 95% CI 0.54–0.81; Meis et al., 2003). Currently, offering 250 mg intramuscular 17-OHPC weekly from 16 to 36 weeks gestation to women with a singleton pregnancy and a history of a prior singleton SPTB <37 weeks is standard of care in the United States (Committee on Practice Bulletins-Obstetrics and The American College of Obstetricians and Gynecologists, 2012; Society for Maternal-Fetal Medicine Publications Committee, with assistance of Vincenzo Berghella, 2012). Unfortunately, 17-OHPC is only effective for some women and as many as 30–40% will experience a recurrent SPTB despite treatment. The mechanism of action of 17-OHPC has not yet been elucidated.

### Assessing reasons for variable response to 17-OHPC

Several recent studies have focused on defining which individuals are destined to have a favorable response to progesterone for the prevention of recurrent preterm birth. These investigations have identified several distinct and consistent clinical risk factors for recurrent SPTB despite 17-OHPC therapy, including black race, gonorrhea or chlamydia infection, vaginal bleeding, family history of SPTB, male fetus (Manuck et al., 2016a,c). Pharmacogenomic studies assessing recurrent SPTB among women using 17-OHPC have been pre-clinical in nature, but have also shown promising results, implicating biologically plausible pathways including the nitric oxide pathway and other genetic pathways involved with signal transduction and infection and inflammation (Manuck et al., 2014b; Manuck, 2016). Unfortunately, insufficient sample numbers, difficulties in defining adequate treatment “response” and lack of randomized studies has prevented this work from reaching direct clinical applicability (Manuck, 2016). Studies examining more acute genetic changes (e.g., epigenetic modifications) in response to the environmental stimuli of 17-OHPC are also currently limited but may offer additional avenues of research and eventual clinical application. Finally, it is possible that genotype or methylation changes affect birth outcomes only in the presence of a certain critical threshold or serum level of 17-OHPC. Many drugs taken by women for a variety of indications during pregnancy are metabolized by the cytochrome P450 enzyme system; 17-OHPC is also metabolized by the cytochrome P450 system, primarily CYP3A (Sharma et al., 2010). Interactions with co-administered CYP3A inhibitors or inducers may influence 17-OHPC drug metabolism and clinical efficacy (Sharma et al., 2008). No studies examining the effects of concomitant drug administration with 17-OHPC have been published to date, though *in vitro* data suggests these other CYP-metabolized drugs may impact 17-OHPC levels (Sharma et al., 2008).

### Using state-of-the-art genetic technology to investigate other reasons for variable outcomes

Epigenetic modifications provide a mechanism by which genes interact with the environment; they affect gene expression by inducing structural changes in DNA that are maintained through cell division, respond to environmental changes including drug exposures, yet are potentially reversible and can be targets for disease therapy (Feinberg, 2007). DNA methylation at cytosine-guanine dinucleotides sites, the most commonly studied epigenetic modification in humans, guides temporal, and tissue-specific gene expression during fetal development and tissue differentiation. Even subtle environmental changes may induce epigenetic changes and have effects on phenotype (Golbabapour et al., 2011). Epigenetic biomarkers from blood have been used in a variety of complex conditions in other fields (e.g., depression, addiction/smoking). Moreover, these changes are potentially reversible and reflect dynamic interactions with the environment. For example, studies of women with breast cancer (Antoni et al., 2012; Bower et al., 2014; Stagl et al., 2015), caregivers of family members with dementia (Black et al., 2013), patients with inflammatory bowel disease (Kuo et al., 2015), and even healthy individuals (Qu et al., 2013) have shown that the body's response to chronic stress may be dynamic, mediated by glucocorticoid gene expression, and at least partly reversible **at the genomic level** when treated with cognitive behavioral therapy or relaxation techniques. Therefore, study of the methylome and transcriptome is a promising and understudied avenue of investigation in prematurity research (Fuchikami et al., 2011; Mikeska et al., 2012; Philibert et al., 2013). Methylation is tissue specific; results from blood, placental, or cervical tissue, for example typically cannot be directly compared. However, methylation studies in blood may be a reliable correlate of physiologic processes in other tissues (Smith et al., 2014). Limited studies of epigenetics in obstetrics have demonstrated identifiable differences among women delivering preterm and those with term deliveries, but more work is needed to evaluate whether these differences may be influenced (positively or negatively) by medication exposure or other known risks factors for SPTB, such as vaginal bleeding or a short cervical length.

### Refining clinical phenotype to improve preterm birth outcome predictions

Unfortunately, the aforementioned pharmacogenomics associations have been modest. Larger scale genome wide association studies of SPTB in general (without considering treatment response) have also been largely disappointing. It has been hypothesized that suboptimal clinical phenotyping is a major contributing factor to the challenges of reproducibility that have characterized genetic investigations of prematurity. Many genetic studies of SPTB have limited or inconsistent phenotype information; even those that do collect detailed information tend to be based on relatively heterogeneous groups of women. We recently described a phenotype classification system. This tool uses clinical data to group women into nine distinct SPTB phenotypes (e.g., cervical insufficiency, infection/inflammation, decidual hemorrhage), and may provide further insight beyond the heterogenous PTB categorization, improving upon prior classification systems (Manuck et al., 2015).

## Treatment of acute spontaneous preterm labor with indomethacin

One of the few tocolytic medications proven to prolong pregnancy is indomethacin, a non-steroidal anti-inflammatory medication that inhibits prostaglandin production by blocking the conversion of arachidonic acid to prostaglandin. Indomethacin is metabolized by CYP2C9 and carboxy esterase (Agúndez et al., 2009). Cells in the amnion, chorion, decidua, and myometrium exhibit enhanced prostaglandin production in response to cytokines interleukin-1β, interleukin-6, and tumor necrosis factor-α, and may help initiate the parturition cascade (Romero et al., 1989a,b; Mitchell et al., 1990). It is therefore hypothesized that indomethacin has a direct effect in the inhibition of the inflammatory response. Indomethacin has been shown to be more effective than placebo in prolonging pregnancies with threatened preterm labor by >48 h, and use for women with acute preterm labor may permit administration of a course of antenatal corticosteroids. Despite this, indomethacin has not been proven to improve short- or long- term neonatal outcomes (Niebyl et al., 1980; Panter et al., 1999; Klauser et al., 2014).

Though somewhat effective, indomethacin is not without potential risk to the fetus, and these risks have limited its use. Indomethacin freely crosses the placenta and enters fetal circulation. Premature closure of the physiologically patent ductus arteriosus is a recognized side effect of indomethacin and is the leading reason why tocolysis with indomethacin is generally limited to short courses of therapy (48–72 h; Moise, 1993). Some studies suggest that neonates exposed to antenatal indomethacin may have an increased risk of other adverse effects including periventricular leukomalacia and necrotizing enterocolitis (Major et al., 1994; Amin et al., 2007), although results have been somewhat inconsistent between studies. (Dudley and Hardie, 1985; Macones and Robinson, 1997; Loe et al., 2005) Despite these safety concerns, indomethacin is one of the first line recommended tocolytics by the American Congress of Obstetricans and Gynecologists, and short courses of indomethacin are widely used in the US for acute tocolysis due to its relative effectiveness compared to other agents (American College of Obstetricans and Gynecologists' Committee on Practice Bulletins-Obstetrics, 2016).

### Assessing reasons for variable response to indomethacin

Determination of individuals most likely to remain pregnant 48 h after indomethacin therapy is a challenge, as placebo-controlled tocolytic studies are limited and retrospective studies are fraught with confounding. The diagnosis of “preterm labor” remains imperfect, and studies have traditionally used varying definitions to determine participant eligibility. Many women initially diagnosed with acute preterm labor will eventually deliver at term. We are unaware of any reported studies of indomethacin pharmacogenetics in the setting of pregnancy, though investigation of maternal and/or fetal genome, epigenome, methylome may shed additional light on variation in response in a similar fashion to pharmacogenomics investigations of 17-OHPC as outlined above.

### Application of nanomedicine to target indomethacin delivery to gestational tissues

If women who are most likely to benefit from indomethacin treatment for acute preterm labor can be identified through pharmacogenomic or other investigations, the issue of potential toxicity to the fetus must be resolved in order for this therapy to be administered optimally. Recently, nanomedicine has been investigated as one potential way to overcome this limitation. Nanomedicine provides a mechanism to vector drugs (e.g., through liposomes) preferentially to diseased or target tissues in the body, while limiting exposure (and thus toxicity) to healthy tissues (and in the case of pregnancy, the fetus). Specifically engineered liposomes, encapsulated with indomethacin and with a surface oxytocin receptor antagonist, have been designed for this purpose, to deliver indomethacin directly to the myometrium while limiting fetal exposure. In initial murine studies, these liposomes successfully delivered indomethacin to the uterus and inhibited prostaglandin production—thus maintaining its pharmacologic effects—while reducing fetal exposure by 7.6-fold. (Refuerzo et al., 2015) In subsequent murine models, these specifically engineered liposomes prolonged pregnancy by 31% and reduced the rate of PTB by 15% (Refuerzo et al., 2016). The application of nanotechnology has great promise as a solution to the fetal toxicity appreciated with indomethacin, and refinement of these techniques will expand researcher's abilities to investigate other therapeutics for SPTB prevention and treatment with lower concern for fetal exposure.

## Summary

Preterm birth is a devastating obstetric complication leading to fetal mortality and morbidity. Despite extensive research, there remains much to learn regarding the pathophysiological mechanisms associated with SPTB. We understand even less about the role that epigenetic regulation and subsequent altered gene expression play as an etiologic factor for this condition. Progestogens and tocolytic drugs can reduce neonatal morbidity and mortality by preventing or halting SPTB through poorly defined mechanisms. Additional individual patient characteristics, including maternal and fetal genotype and gene-environment interactions likely influence response. Other factors, beyond the scope of this article—such as cytochrome P450 enzyme activity, maternal body mass index, and other considerations that may impact volume of drug distribution may also impact response to medications for SPTB. Studies of all SPTB therapeutics should collect and incorporate rigorous clinical phenotype information and biologic sample data whenever possible, in order for further refine and integrate clinical phenotype, genotype, and response to preventative and therapeutic medications.

## Author contributions

The author confirms being the sole contributor of this work and approved it for publication.

### Conflict of interest statement

The author declares that the research was conducted in the absence of any commercial or financial relationships that could be construed as a potential conflict of interest.
